# ReDO_DB: the repurposing drugs in oncology database

**DOI:** 10.3332/ecancer.2018.886

**Published:** 2018-12-06

**Authors:** Pan Pantziarka, Ciska Verbaanderd, Vidula Sukhatme, I Rica Capistrano, Sergio Crispino, Bishal Gyawali, Ilse Rooman, An MT Van Nuffel, Lydie Meheus, Vikas P Sukhatme, Gauthier Bouche

**Affiliations:** 1 The Anticancer Fund, Brussels, 1853 Strombeek-Bever, Belgium; 2 The George Pantziarka TP53 Trust, London, UK; 3 Clinical Pharmacology and Pharmacotherapy, Department of Pharmaceutical and Pharmacological Sciences, KU Leuven, Leuven, Belgium; 4 GlobalCures Inc., Newton, MA 02459 USA; 5 Department of Medicine, Brigham and Women’s Hospital, Harvard Medical School, Boston, MA 02115 USA; 6 Oncology Research Centre, Vrije Universiteit Brussel, Brussels, Belgium; 7 Emory University School of Medicine, Atlanta, GA 30322 USA

**Keywords:** drug repurposing, repositioning, ReDO project, cancer drugs, online database

## Abstract

Repurposing is a drug development strategy that seeks to use existing medications for new indications. In oncology, there is an increased level of activity looking at the use of non-cancer drugs as possible cancer treatments. The Repurposing Drugs in Oncology (ReDO) project has used a literature-based approach to identify licensed non-cancer drugs with published evidence of anticancer activity. Data from 268 drugs have been included in a database (ReDO_DB) developed by the ReDO project. Summary results are outlined and an assessment of clinical trial activity also described. The database has been made available as an online open-access resource (http://www.redo-project.org/db/).

## Introduction

Drug repurposing, also known as repositioning, is a strategy that seeks new medical treatments from among existing licensed medications rather than from the development of new molecules (*de novo* drug development) [1]. Repurposing is by no means a new idea in medicine, indeed, many venerable and well-established drugs, for example, the beta-blocker propranolol, have been extensively repurposed many times in the past. However, as an explicit development strategy, repurposing is being increasingly pursued in a number of different disease areas [2–4]. Indeed, data from PubMed show that the number of publications related to drug repurposing or repositioning has increased exponentially since 2004 [5].

The Repurposing Drugs in Oncology (ReDO) project is an on-going collaborative project that has focused exclusively on the potential use of licensed non-cancer medications as sources of new cancer therapeutics [6]. While it is a common practice for new cancer medicines to be licensed for additional cancer indications after an initial license has been granted, a process that has been termed ‘soft repurposing’, the licensing of non-cancer medications as new treatments is relatively uncommon, hence this process has been termed ‘hard repurposing’ [7]. Indeed, there are very few examples in standard clinical practice of non-cancer drugs being moved into oncology, with thalidomide (multiple myeloma) and all-trans retinoic acid (acute promyelocytic leukaemia) being the best-known examples. In this sense, the ReDO project has focused exclusively on hard drug repurposing in oncology.

In contrast to *de novo* drugs, licensed medications may offer a number of advantages in terms of development [8]:

- Availability of pharmacokinetics, pharmacodynamics and posology data
- Knowledge of safety and toxicity, including rare adverse events
- Clinical experience derived from the original indications
- Widespread availability—particularly for drugs included in the WHO Essentials Medicines list (EML)
- Low cost—particularly for generic medications with multiple manufacturers
- Understanding of mechanisms of action and/or molecular targets

However, while these advantages may shorten the development period in comparison to unlicensed medications, particularly with respect to early phase toxicity trials, proving efficacy in any new indications remains a challenge. Despite the advantages that repurposed drugs may offer in terms of toxicity and cost, the single most important criterion by which treatments should be judged is efficacy. Of course, this also means that the medical community should judge the relative merits of repurposed versus new drugs without bias [9].

While much of the burgeoning interest in oncological repurposing is related to a few very high profile candidates, such as aspirin or metformin, there is indeed a wide range of non-cancer drugs which have some level of evidence in support of relevant anticancer activity [10, 11]. This paper introduces ReDO_DB—a database of non-cancer drugs with evidence of anticancer activity that has been developed as part of the ReDO project. In addition to outlining the methodology and a selection of results from the database, it also gives details of the online open access publication of the database so that the data can be freely used by clinicians and researchers interested in developing specific repurposing projects.

## Methodology

The ReDO project has adopted a literature-based methodology to identify non-cancer drugs with anticancer potential. The academic literature was actively scanned and potential repurposing candidates identified.

### Selection criteria

Potential candidates must match the following criteria:

- The drug is currently licensed for non-cancer indications in at least one country in the world. Not included are:
  - Existing cancer drugs, including cytotoxics, targeted agents or immunotherapeutics, (e.g. docetaxel, cyclophosphamide etc)
  - Drugs withdrawn globally (e.g. phenformin)
  - Experimental medicines or previously shelved compounds (e.g. semagacestat, licofelone etc)
  - Nutraceuticals (e.g. curcumin, resveratrol etc)
- The drug is the subject of one or more peer-reviewed publications showing a specific anticancer effect in one or more malignancies.

The evidence for anticancer effects could come from *in vitro*, *in vivo* or human research (as assessed by performing a PubMed search). *In silico* studies supported with *in vitro* or *in vivo* data were also included.

Drugs are included in the database if they fulfil the criteria above. In some cases, there may be indirect evidence to suggest that a drug may have anticancer activity because it has effects on an oncologically relevant pathway. However, if there is no explicit evidence of an effect—in other words, the evidence is purely mechanistic, then the drug is *not* included in the database.

### Data collected

For each drug added to the database, we recorded its name (international non-proprietary name), synonyms (if relevant) and main approved indications. Each drug was also checked to see if it is available as a generic and whether it is included in the WHO List of Essential Medicines [12]. Multiple data sources were checked to assess whether a drug is available as a generic or is off-patent, although in some cases it was not possible to ascertain the current position with respect to patent protection. We also collected information on the type of research showing the anticancer activity of the drug: *in vitro*, *in vivo* and in humans.

Human data could include individual case reports, case series, epidemiological studies and clinical trials. Case reports were assessed using the following PubMed search terms: *(‘Case Reports’ [Publication Type]) AND cancer AND <drug name>.* Observational studies were assessed using the following PubMed search terms: *(‘Observational Study’ [Publication Type]) AND Cancer AND <drug name>*. Clinical data included published trial reports, of any phase, and existing clinical trial activity (as assessed by checking ClinicalTrials.gov, WHO ICTRP and OpenTrials registries). ReDO_DB was cross-referenced with DrugBank [13, 14] for additional analysis. Data were extracted on the Anatomical Therapeutic Chemical Classification System codes for each drug [15]. Additionally, DrugBank was used to extract data on the validated molecular targets for each drug. Note that these targets are not cancer-specific and as with the ReDO_DB data presented here, the data on molecular targets is a snapshot based on release 5.1.1 of DrugBank (release date 03 July 2018).

Finally, a search was performed to assess clinical trial activity by the drug. Three international clinical trial registries (ClinicalTrials.gov, WHO ICTRP and OpenTrials) were searched for each of the drugs on 16 August 2018. Only active late-stage oncology trials were included—that is trials flagged as Phase 2/3, Phase 3 or Phase 3/4. Each trial was manually assessed to remove trials in which the drugs were being used for their original indication, for example, trials in which licensed antiemetics were being assessed in new cancer or in combination treatments with other antiemetics. For each trial, the following information were collected: drug tested, countries (and continent), sponsor and cancer type.

## Results

### Drugs in the ReDO database

We found a total of 268 drugs that met our selection criteria. In order to maximise the utility of the database, an open access version is made available online via the website of the ReDO project (www.redo-project.org/db). The online version will be periodically updated so that as new drugs are added to the database, or new data become available for existing drugs on the database, the information can be made available to the oncology community. Additions or amendments may also be proposed via an online contact form, enabling other members of the oncology community to contribute to the development of the database. The online database has also been structured in a format to facilitate easy data-mining, spidering or simple cut and paste to maximise accessibility.

The following results summarise the data in the ReDO_DB as of 16 August 2018 when 268 drugs were included. Summary statistics are shown in Table 1.

It should be noted that 25% of drugs meet multiple favourable criteria in that they are on the WHO List of Essential Medicines, are off-patent and have some form of human evidence of anticancer effects.

The complete list of drugs is included in the supplementary data.

Repurposing candidates come from a wide range of areas of medicine. Using the Anatomical Therapeutic Chemical Classification System, we can assess the sources of ReDO_DB drugs, as shown in Table 2. Note that some drugs are included in multiple ATC categories, and therefore the total is greater than the number of drugs in the ReDO dataset.

Data on molecular targets are shown in Table 3.

#### Late stage oncology trials

In all 190 relevant late-stage trials were identified. Data from this analysis are shown in Table 4.

**Figure 1.**
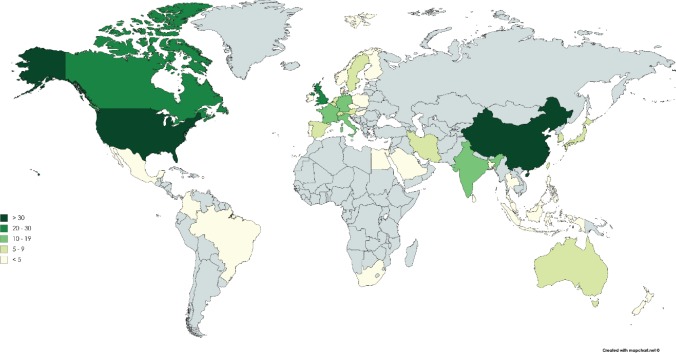
World map showing the number of late-stage trials of the drugs in the ReDO_DB per country.

The characteristics of the 190 trials are summarised in Table 5. Figure 1 shows a map of the countries where the trials have been or are being conducted. A small number of drugs are currently the subject of intense clinical trial activity (i.e. 10 or more active late-stage trials) and should be considered to be well advanced in terms of a ‘repurposing drugs pipeline’ in oncology. In terms of clinical trial sponsorship, the data show that the very few trials have a commercial sponsor—less than 4% of trials in this dataset.

## Discussion

Data from ReDO_DB show that there are in fact a large number of non-cancer drugs with published evidence of anticancer effects. The majority (73%) have some evidence of anticancer effects from case reports, observational studies or clinical trials. Furthermore, the majority (84%) are off-patent, and 32% are included in the WHO EML. The number of drugs which have human data, are off-patent and included in the WHO EML is 67, representing 25% of the total database. This represents a promising pipeline of potential new treatments in oncology. It is indeed encouraging that there are currently just under 200 late-stage clinical trials investigating the use of these drugs in oncology. However, given the high unmet needs in paediatric oncology, it is not so encouraging to note that only 6% of these trials are in childhood cancers.

In terms of molecular drug targets, it is likely that the number of targets reported in Table 3 is an underestimate based on analysis by Mestres *et al* which showed that for a large panel of drugs, the average number of target proteins per drug is 6.3 if additional data sources to DrugBank are accessed [16].

The ReDO_DB has both strengths and limitations. One strength is that the database has been built prospectively over the last 5 years, allowing us to manually curate and validate each drug. We have also benefited from the help of a large network of individuals interested in drug repurposing in oncology. However, we acknowledge that at any given time, the database is incomplete in that new data are published and new candidates emerge. Currently, new entries to the database are added regularly and it is hoped that with the database becoming publicly available, a crowdsourcing effect may help to increase the level of completeness of the database.

ReDO_DB does not include drugs that have solely *in silico* evidence of a possible role in oncology. While our criteria depend on biological data (*in vitro* or *in vivo*) for the inclusion of a candidate drug in the database, we acknowledge the value of *in silico* work as it is often the first method to suggest a brand new use for an existing drug [17]. One limitation of the database is that it does not include existing cancer drugs which represent a large source of repurposing opportunities. However, virtually all cancer drugs are possible candidates for repurposing in other cancer types and listing them in the ReDO_DB would not be of any added value. We also have not included approved vaccines in the database but we are considering doing so, building on the case of Bacillus Calmette–Guérin [18] and looking at recent evidence in support of the repurposing of influenza [19] or cholera vaccine [20].

The inclusion of a number of drugs was problematic in that they are already licensed for use in oncology for symptomatic relief (e.g. aprepitant to control chemotherapy-associated nausea and vomiting) or cancer-related events (e.g. zoledronate or ibandronate for reduction of bone-related events in advanced malignancies). In the former case, drugs are included if there is data to suggest that there is specific anticancer activity independent of the existing licensed indication. With the cases of zoledronate and ibandronate, the issue is complicated in that there is some existing ‘off-label’ use of the drugs for specific anticancer effects. However, as this use is currently off-label, the drugs have been included in the database.

While the inclusion of new drugs in the database is fairly straightforward and is based on the criteria outlined previously, the removal of repurposing candidates is more complex. Failure of a repurposing candidate in a clinical trial is insufficient grounds for removal as the drug may still be active in a different cancer, treatment setting, drug combination or dose. Where a drug has been included based solely on published preclinical work that is later shown to be fraudulent, then removal would be warranted if there is no other supporting evidence. However, the clearest case for removal is when a repurposed drug becomes licensed as a new cancer treatment—in that case, the drug moves into the ‘soft repurposing’ category for further development in other cancer indications and will be removed from the ReDO_DB.

With such a broad range of drugs and so many validated molecular targets, discussion of general mechanisms of action and research priorities is not possible. The vast majority of these drugs do not induce cancer cell cytotoxicity but instead act systemically on the host, alter the immune response or else affect aspects of the tumour microenvironment. These effects may provide therapeutic benefit to cancer patients when used in combination with existing treatments.

The main challenge will be to test these hypotheses to ultimately find cancer indications, if any, for each candidate. For drugs that are already well-studied (e.g. disulfiram or nelfinavir), a meticulous analysis of the data available is needed to identify the most relevant clinical trials to be conducted. For less well-studied drugs (e.g. fasudil or trimetazidine), more research may first be needed to explore and guide the possible future of those drugs. Another possible source of indications for some candidates may be in precision oncology efforts [7].

A number of online cancer-related drug repurposing databases already exist, including DRUGSURV [21], DeSigN [22] and IMPACT [23]. However, these databases are primarily designed to facilitate the discovery of new repurposing candidates using different data sources and algorithmic techniques. In contrast, ReDO_DB presents a curated list of repurposing candidates and a summary of the types of data sources supporting the inclusion of the drug in the database. Outside of oncology, the PDE3 (Prescribable Drugs with Efficacy in Experimental Epilepsies) [2] is an example of a database similar in scope and intention to ReDO_DB.

There is clearly a scope to increase the value of the database in the future by the inclusion of additional data fields. One possibility is to include an indication of the strength of evidence for each of the drugs in addition to showing the range (*in vitro*, *in vivo* etc) of evidentiary sources. Other enhancements may also be proposed by users of the database in the future.

## Conclusion

The results outlined in this paper are generally positive in showing both a growth of interest in drug repurposing, a wide range of candidates for repurposing in oncology and 190 late-stage clinical trials. However, it is also true that there are numerous obstacles in the path to successful repurposing. Because many of the repurposing candidates are generic drugs, (84% in the ReDO dataset), commercial funding of clinical trials is normally not an option. Indeed, the data in Table 5 show that only 7 of 190 trials were sponsored by pharmaceutical companies.

There are significant costs associated with carrying out large Phase III efficacy trials—repurposing trials are therefore at a disadvantage in that they must rely on state or philanthropic sources of funding. Indeed, there is even some evidence to suggest that for an institution there is a financial benefit from running a commercial trial (i.e. per patient net income) compared to a non-commercial trial (i.e. per patient net cost) [24, 25]. In some cases, there may be commercial support for repurposing trials if a commercial sponsor is looking to increase efficacy or expand an indication for an on-patent drug by combining with a repurposing candidate. There may also be cases where insurers or other payers may wish to fund studies that have the potential to reduce cancer recurrence rates or other interventions designed to reduce their costs. Finally, the costs of studies using repurposed drugs may fall if suitable biomarkers are used to stratify patients for enrolment who are most likely to benefit, thereby reducing patient numbers required to show an effect.

Here again repurposing faces a financial obstacle in that there are costs associated with licensing a drug for a new indication (technically, a label extension). It is also the case that there are regulatory restrictions on who can apply for a label extension—therefore, it is important to make the case for a ‘public benefit label extension’ process so that we can move clinically-proven repurposing from ‘off-label’ to ‘on-label’ treatments [26]. In time, we hope that we will see the ReDO_DB shrink as repurposed drugs are licensed for new cancers indications, at which point they will be removed from the database.

## Competing interests

The authors declare that they have no competing interests. All the authors are associated with not for profit organisations that aim to repurpose drugs for oncology treatments. VPS is also a scientific advisory board member of Berg Health and Mitra Biotech.

## Figures and Tables

**Table 1.** Summary statistics from ReDO_DB as of 15 August 18.

Drugs …	Yes	%	No	%	Total
Are included in WHO List of Essential Medicines?	87	32	181	68	268
Are off-patent? §	226	84	35	13	268
Are supported by *in vitro* evidence?	264	99	4	1	268
Are supported by *in vivo* evidence?	247	92	21	8	268
Are supported by case reports?	86	32	182	68	268
Are supported by observational studies?	36	13	232	87	268
Have been/Are being tested in clinical trials?	178	66	90	34	268
Have trial report(s) published?	113	63	65	37	178
Are supported by human data? *	194	72	74	28	268
Are WHO + Off-patent + Human data?*	67	25	201	75	268

* At least one case report, observational study or clinical trial.

§ It was not possible to ascertain the patent position for 3% of drugs.

**Table 2.** Number of drugs by top-level ATC category.

ATC Level 1	Drugs
Cardiovascular System	56
Nervous System	49
Alimentary Tract and Metabolism	39
Musculo-Skeletal System	31
Antiinfectives for Systemic Use	26
Dermatologicals	23
Genito Urinary System and Sex Hormones	23
Sensory Organs	22
Antiparasitic Products, Insecticides and Repellents	20
Blood and Blood-Forming Organs	16
Antineoplastic and Immunomodulating Agents	12
Respiratory System	11
Systemic Hormonal Preparations, Excl. Sex Hormones and Insulins	4
Various	4

**Table 3.** Molecular targets included in ReDO drugs.

Item	
ReDO drugs included in DrugBank*	263
ReDO drugs with targets in DrugBank	252
Total targets identified in all ReDO drugs	1201
Number of unique targets in ReDO drugs	660
Average targets per drug	4.77

* Five drugs approved for use outside of the USA and the EU are not currently included in DrugBank.

**Table 4.** ReDO drugs included in late-phase clinical trials.

Item	
Number of relevant late-stage trials	190
Number of unique drugs	72
Number of drugs with 5 or more trials	11
Number of drugs with 10 or more trials	6

**Table 5.** Characteristics of the 190 trials registered with one of the 72 drugs of the ReDO_DB tested in clinical trials.

		*N*	%
**Drug with more than 10 trials**			
	Acetylsalicylic acid	27	14
	Celecoxib	12	6
	Cholecalciferol	12	6
	Metformin	17	9
	Olanzapine	10	5
	Zoledronic Acid	20	11
**Cancer Type**			
	Gastrointestinal	53	28
	Breast	38	20
	Hematologic	23	12
	Lung	14	7
	Gynaecologic	11	6
	Brain and CNS	10	5
	Other	24	13
	Paediatric	12	6
	Not specified	23	12
**Trial Location**			
	Europe	68	36
	Asia	61	32
	North America	48	25
	Middle East	11	6
	Oceania	7	4
	South & Central America	6	3
	Africa	5	3
**Sponsor**			
	University and/or hospital	127	67
	Research Institute, organisation, foundation or network	53	28
	Small- and medium-sized pharmaceutical companies	6	3
	Government	3	2
	Large pharmaceutical companies	1	1

## Supplementary: ReDO drugs list— 16 August 2018

**Table:**

Drug	Synonym	Main Indications	WHO	Off-Patent	Vitro	Vivo	Cases	Obs. Studies	Trials	Trial Report	Human data
Acetaminophen	Paracetamol	Analgesia	Yes	Yes	Yes	Yes	Yes	Yes	Yes	Yes	Yes
Acetazolamide		Glaucoma, diuretic, epilepsy	Yes	Yes	Yes	Yes	No	No	Yes	No	Yes
Acetylsalicylic acid	Aspirin	Analgesia, swelling, prophylaxis of venous embolism and further heart attacks or strokes	Yes	Yes	Yes	Yes	No	Yes	Yes	Yes	Yes
Agomelatine		Insomnia	No	No	Yes	No	No	No	No	No	No
Albendazole		Parasitic infection	Yes	Yes	Yes	Yes	Yes	No	Yes	No	Yes
Alendronic Acid	Alendronate	Osteoporosis	No	Yes	Yes	Yes	Yes	No	Yes	Yes	Yes
Aliskiren		Essential hypertension	No	No	Yes	No	No	No	No	No	No
Allopurinol		Gout	Yes	Yes	Yes	Yes	No	No	Yes	Yes	Yes
Alpha-Lipoic Acid	Thioctic Acid, Lipoic Acid	Diabetic neuropathy (Germany)	No	Yes	Yes	Yes	Yes	No	Yes	Yes	Yes
Amantadine		Parkinson’s Disease, Influenza A	No	Yes	Yes	No	No	No	No	No	No
Amiloride		In congestive heart failure or hypertension treated with thiazides, to conserve potassium	Yes	Yes	Yes	Yes	No	No	No	No	No
Amiodarone		Ventricular tachycardia/fibrillation	Yes	Yes	Yes	Yes	No	No	Yes	Yes	no
Amitriptyline		Depression	Yes	Yes	Yes	Yes	No	No	No	No	No
Amlodipine		Hypertension	Yes	Yes	Yes	Yes	No	No	No	No	No
Amodiaquine		Malaria	Yes	Yes	Yes	No	No	No	No	No	No
Anagrelide		Essential thrombocythemia	No	No	Yes	Yes	No	No	No	No	No
Anakinra		RA, NOMID, CAPS	No	No	Yes	Yes	Yes	No	Yes	Yes	Yes
Aprepitant		Nausea, vomiting	No	Yes	Yes	Yes	No	No	No	No	No
Aprotinin		Perioperative blood loss	No	No	Yes	Yes	No	No	Yes	Yes	Yes
Aripiprazole		Bipolar disorder, major depressive disorder, authistic disorder	No	Yes	Yes	No	No	No	No	No	No
Artesunate		Malaria	Yes	Yes	Yes	Yes	Yes	No	Yes	Yes	Yes
Ascorbic acid	Ascorbate, Vitamin C	Scurvy	Yes	Yes	Yes	Yes	Yes	No	Yes	Yes	Yes
Atenolol		Hypertension, angina pectoris	No	Yes	Yes	No	No	No	No	No	No
Atorvastatin		Coronary heart disease, acute coronary syndrome	No	Yes	Yes	Yes	No	Yes	Yes	Yes	Yes
Atovaquone		Pneumocystis carinii pneumonia, toxoplasmose	No	Yes	Yes	Yes	No	Yes	Yes	No	Yes
Atrial Natriuretic Peptide	Carperitide	Heart failure	No	No	Yes	Yes	No	Yes	Yes	No	Yes
Auranofin		RA	No	Yes	Yes	Yes	No	No	Yes	No	Yes
Azithromycin		Bacterial infection, CAP, PID	Yes	Yes	Yes	Yes	No	No	Yes	Yes	Yes
Bazedoxifene		Osteoporosis	No	No	Yes	Yes	No	No	Yes	No	Yes
Bedaquiline		Tuberculosis	Yes	No	Yes	No	No	No	No	No	No
Bemiparin		Venous thromboembolism, myocardial infarction	No	No	Yes	Yes	No	No	Yes	Yes	Yes
Benserazide		Parkinson’s Disease	No	Yes	Yes	Yes	No	No	No	No	No
Benztropine	Benzatropine	Parkinson’s Disease	No	Yes	Yes	Yes	No	No	No	No	No
Bepridil		Hypertension and chronic stable angina	No	Yes	Yes	Yes	No	No	No	No	No
Bezafibrate		Hyperlipidaemia	No	Yes	Yes	Yes	No	No	Yes	Yes	Yes
Biperiden		Parkinson’s Disease	Yes	Yes	Yes	Yes	No	No	No	No	No
Bosentan		PAH	No	Yes	Yes	Yes	No	No	Yes	Yes	Yes
Bromocriptine		Parkinson’s Disease, prevention of lactation	No	Yes	Yes	Yes	No	No	Yes	Yes	Yes
Cabergoline		Hyperprolactinaemia	No	Yes	Yes	Yes	No	No	Yes	Yes	Yes
Caffeine		Newborn apnoea	No	Yes	Yes	Yes	Yes	No	Yes	Yes	Yes
Calcitriol	Vitamin D3	Vitamin D deficiency	No	Yes	Yes	Yes	Yes	No	Yes	Yes	Yes
Canagliflozin		Diabetes	No	No	Yes	Yes	No	No	No	No	No
Candesartan		Hypertension	No	Yes	Yes	Yes	No	No	Yes	No	Yes
Captopril		Hypertension	No	Yes	Yes	Yes	No	No	Yes	No	Yes
Carbimazole		Hyperthyroidism	No	Yes	Yes	No	No	No	No	No	No
Carglumic Acid		Hyperammonaemia in N-acetylglutamate synthase deficiency	No	No	Yes	Yes	No	No	No	No	No
Carvedilol		Hypertension	No	Yes	Yes	Yes	No	No	Yes	No	Yes
Celecoxib		OA, RA, JRA, AS, acute pain, primary dysmenorrhoea	No	Yes	Yes	Yes	Yes	No	Yes	Yes	Yes
Cephalexin		Bacterial infections	No	Yes	Yes	Yes	No	No	Yes	No	Yes
Chloramphenicol		Superficial eye infections, typhoid fever	Yes	Yes	Yes	No	Yes	No	No	No	Yes
Chloroquine		Malaria, Extraintestinal Amoebiasis	Yes	Yes	Yes	Yes	No	No	Yes	No	Yes
Chlorpromazine		Psychotic disorders, nausea and vomiting, anxiety, hiccups	Yes	Yes	Yes	Yes	No	No	Yes	No	Yes
Cholecalciferol	Colecalciferol, Vitamin D3	Vitamin D deficiency	Yes	Yes	Yes	Yes	Yes	No	Yes	No	Yes
Ciclopirox		Athlete’s foot, ringworm	No	Yes	Yes	Yes	No	No	Yes	Yes	Yes
Cidofovir		CMV-retinitis in AIDS	No	Yes	Yes	Yes	No	No	Yes	Yes	Yes
Cilnidipine		Hypertension	No	Yes	Yes	Yes	No	No	No	No	No
Cimetidine		Duodenal/gastric ulcers, GERD, pathological hypersecretory conditions	No	Yes	Yes	Yes	Yes	No	Yes	Yes	Yes
Ciprofloxacin		Antibiotic	Yes	Yes	Yes	Yes	No	No	Yes	Yes	Yes
Citalopram		Depression	No	Yes	Yes	Yes	No	No	Yes	No	Yes
Clarithromycin		Bacterial infections	Yes	Yes	Yes	Yes	Yes	No	Yes	Yes	Yes
Clodronic acid	Clodronate	Osteolytic lesions, hypercalcaemia and bone pain associated with skeletal metastases in patients with breast cancer or multiple myeloma	No	Yes	Yes	Yes	Yes	No	Yes	Yes	Yes
Clofoctol		Bacterial infections	No	Yes	Yes	Yes	No	No	No	No	No
Clomifene		Ovulatory dysfunction	Yes	Yes	Yes	Yes	No	No	Yes	No	Yes
Clomipramine		Obsessive Compulsive Disorder	Yes	Yes	Yes	Yes	No	No	No	No	No
Clopidogrel		Stroke, post-myocardial infarction	Yes	Yes	Yes	Yes	No	No	Yes	Yes	Yes
Clotrimazole		Fungal infections	Yes	Yes	Yes	Yes	No	No	No	No	No
Colchicine		Gout	No	Yes	Yes	Yes	No	No	Yes	No	Yes
Dalteparin		DVT (prophylaxis), unstable angina/non-Q-wave myocardial infarction	No	Yes	Yes	Yes	Yes	No	Yes	Yes	Yes
Danazol		Endometriosis, fibrocystic breast disease, hereditary angioedema	No	Yes	Yes	Yes	No	No	Yes	Yes	Yes
Dapsone		Dermatitis herpetiformis, leprosy	Yes	Yes	Yes	Yes	No	No	Yes	No	Yes
Deferasirox		Acute iron intoxication, chronic iron overload	No	No	Yes	Yes	No	No	Yes	Yes	Yes
Deferiprone		Iron overload in thalassaemia major	No	No	Yes	Yes	No	No	No	No	No
Deferoxamine	Desferrioxamine	Acute iron intoxication, chronic iron overload	Yes	Yes	Yes	Yes	No	No	Yes	Yes	Yes
Desmopressin		Diabetes Insipidus, bedwetting, haemophilia A, von Willebrand’s disease	Yes	Yes	Yes	Yes	No	No	Yes	No	Yes
Diclofenac		OA, RA, AS	No	Yes	Yes	Yes	Yes	No	Yes	Yes	Yes
Diflunisal		OA, RA, mild to moderate pain	No	Yes	Yes	Yes	No	No	No	No	No
Digitoxin		Congestive HF, atrial fibrillation, atrial flutter, PAT, cardiogenic shock	No	Yes	Yes	Yes	No	No	Yes	No	Yes
Digoxin		Heart failure, atrial fibrillation	Yes	Yes	Yes	Yes	No	No	Yes	No	Yes
Dimethyl Fumarate		Psoriasis, Multiple Sclerosis	No	Yes	Yes	Yes	No	No	Yes	No	Yes
Dipyridamole		Thromboembolism Prophylaxis Post-Cardiac Valve Replacement	No	Yes	Yes	Yes	No	No	Yes	No	Yes
Disulfiram		Chronic alcoholism	No	Yes	Yes	Yes	No	No	Yes	Yes	Yes
Donepezil		Alzheimer’s Disease	No	Yes	Yes	Yes	No	No	Yes	Yes	Yes
Doxazosin		Hypertension, benign prostatic hyperplasia	No	Yes	Yes	Yes	No	No	No	No	No
Doxycycline		Respiratory/urinary tract/ophthalmic infection	Yes	Yes	Yes	Yes	Yes	No	Yes	Yes	Yes
Dutasteride		Benign prostatic hyperplasia	No	Yes	Yes	Yes	Yes	No	Yes	Yes	Yes
Ebastine		Allergies	No	Yes	Yes	Yes	No	No	No	No	No
Efavirenz		Anti-retroviral	Yes	Yes	Yes	Yes	No	No	Yes	Yes	Yes
Eflornithine	DFMO	Adjunct to laser therapy for facial hirsutism in women, African trypanosomiasis	Yes	Yes	Yes	Yes	Yes	No	Yes	Yes	Yes
Enalapril		Hypertension	Yes	Yes	Yes	Yes	No	No	Yes	No	Yes
Enoxaparin		Prophylaxis of venous thromboembolism	Yes	Yes	Yes	Yes	No	No	Yes	Yes	Yes
Epalrestat		Diabetes	No	Yes	Yes	Yes	No	No	Yes	No	Yes
Esomeprazole		Antacid	No	Yes	Yes	Yes	Yes	Yes	Yes	Yes	Yes
Ethacrynic Acid	Etacrynic acid	Diuretic	No	Yes	Yes	Yes	Yes	No	No	No	Yes
Etodolac		Analgesia	No	Yes	Yes	Yes	Yes	No	Yes	Yes	Yes
Famotidine		Antacid	No	Yes	Yes	Yes	No	No	Yes	Yes	Yes
Fasudil		Vasodilator	No	Unclear	Yes	Yes	No	No	No	No	No
Felodipine		Hypertension	No	Yes	Yes	Yes	No	No	No	No	No
Fenofibrate		Hyperlipidaemia	No	Yes	Yes	Yes	Yes	No	Yes	Yes	Yes
Finasteride		Benign prostatic hyperplasia	No	Yes	Yes	Yes	Yes	Yes	Yes	Yes	Yes
Fingolimod		Multiple Sclerosis	No	No	Yes	Yes	No	No	Yes	No	Yes
Flubendazole		Parasitic infection	No	Yes	Yes	Yes	No	No	No	No	No
Flucytosine	5-Fluorocytosine	Candida and/or Cryptococcus	Yes	Yes	Yes	Yes	No	No	Yes	Yes	Yes
Fluoxetine	Prozac	Depression	Yes	Yes	Yes	Yes	No	No	No	No	No
Fluspirilene		Psychotic disorders	No	Yes	Yes	Yes	No	No	No	No	No
Flurbiprofen		Analgesia	No	Yes	Yes	Yes	No	Yes	Yes	Yes	Yes
Fluvastatin		Hyperlipidaemia	No	Yes	Yes	Yes	Yes	Yes	Yes	Yes	Yes
Fluvoxamine		Depression	No	Yes	Yes	Yes	Yes	No	No	No	Yes
Ganciclovir		Anti-viral	No	Yes	Yes	Yes	Yes	No	Yes	Yes	Yes
Glipizide		Diabetes	No	Yes	Yes	Yes	No	No	No	No	No
Glibenclamide	Glyburide	Diabetes	No	Yes	Yes	Yes	No	No	No	No	No
Griseofulvin		Fungal infections	Yes	Yes	Yes	Yes	Yes	No	No	No	Yes
Haloperidol		Psychotic disorders	Yes	Yes	Yes	Yes	No	No	No	No	No
Hydralazine		Hypertension	Yes	Yes	Yes	Yes	No	No	Yes	Yes	Yes
Hydroxychloroquine		Malaria	Yes	Yes	Yes	Yes	Yes	No	Yes	Yes	Yes
Hymecromone		Antispasmodic	No	Yes	Yes	Yes	No	No	Yes	No	Yes
Ibandronic acid	Ibandronate	Osteoporosis	No	Yes	Yes	Yes	Yes	No	Yes	No	Yes
Ibuprofen		Analgesia	Yes	Yes	Yes	Yes	No	No	Yes	No	Yes
Imipramine		Depression	No	Yes	Yes	Yes	No	No	Yes	No	Yes
Indomethacin	Indometacin	Analgesia	No	Yes	Yes	Yes	Yes	No	Yes	Yes	Yes
Irbesartan		Hypertension	No	Yes	Yes	Yes	Yes	Yes	No	No	Yes
Itraconazole		Fungal infections	Yes	Yes	Yes	Yes	Yes	Yes	Yes	Yes	Yes
Ivermectin		Parasitic infection	Yes	Yes	Yes	Yes	No	No	Yes	No	Yes
Ketamine		Anaesthetic	Yes	Yes	Yes	Yes	No	No	Yes	No	Yes
Ketoconazole		Fungal infections	No	Yes	Yes	Yes	Yes	Yes	Yes	Yes	Yes
Ketorolac		Post-operative analgesia	No	Yes	Yes	Yes	Yes	Yes	Yes	Yes	Yes
Lamotrigine		Epilepsy	Yes	Yes	Yes	Yes	No	No	No	No	No
Lansoprazole		Antacid	No	Yes	Yes	Yes	No	Yes	Yes	No	Yes
L-Arginine		Nutraceutical	No	Yes	Yes	Yes	No	No	Yes	No	Yes
Leflunomide		Arthritis	No	Yes	Yes	Yes	No	No	Yes	Yes	Yes
Levetiracetam		Epilepsy	No	Yes	Yes	No	Yes	Yes	Yes	No	Yes
Levofloxacin		Antibiotic	Yes	Yes	Yes	Yes	Yes	No	No	No	Yes
Levonorgestrel		Contraceptive	Yes	Yes	Yes	Yes	Yes	Yes	Yes	Yes	Yes
L-Glutamine		Nutraceutical	No	Yes	Yes	Yes	No	No	Yes	Yes	Yes
Lidocaine		Anaesthetic	Yes	Yes	Yes	Yes	No	No	Yes	No	Yes
Lithium		Bipolar disorders	Yes	Yes	Yes	Yes	Yes	No	Yes	No	Yes
Loperamide		Diabetes	Yes	Yes	Yes	No	No	No	No	No	No
Lopinavir		Anti-retroviral	Yes	Yes	Yes	Yes	No	No	Yes	Yes	Yes
Loratadine		Allergies	Yes	Yes	Yes	No	No	Yes	No	No	Yes
Losartan		Hypertension	Yes	Yes	Yes	Yes	No	Yes	Yes	No	Yes
Lovastatin		Hyperlipidaemia	No	Yes	Yes	Yes	Yes	No	Yes	Yes	Yes
Loxoprofen		Analgesia	No	Yes	Yes	Yes	Yes	Yes	No	No	Yes
Macitentan		Pulmonary arterial hypertension	No	No	Yes	Yes	No	No	Yes	No	Yes
Manidipine		Hypertension	No	Yes	Yes	Yes	No	No	No	No	No
Maraviroc		Anti-retroviral	No	No	Yes	Yes	No	No	Yes	Yes	Yes
Mebendazole		Parasitic infection	Yes	Yes	Yes	Yes	Yes	No	Yes	No	Yes
Meclofenamate	Meclofenamic acid	Analgesia	No	Yes	Yes	Yes	No	No	Yes	No	Yes
Mefloquine		Malaria	Yes	Yes	Yes	Yes	No	No	Yes	No	Yes
Megestrol Acetate		Hormone	No	Yes	Yes	Yes	Yes	No	Yes	Yes	Yes
Melatonin		Insomnia	No	Yes	Yes	Yes	Yes	No	Yes	Yes	Yes
Meloxicam		Analgesia	No	Yes	Yes	Yes	Yes	No	Yes	Yes	Yes
Memantine		Alzheimer’s Disease	No	Yes	Yes	No	No	No	Yes	Yes	Yes
Mepacrine	Quinacrine	Parasitic infection	No	Yes	Yes	Yes	No	No	Yes	Yes	Yes
Mesalazine	Mesalamine, 5-aminosalicylic acid	Inflammatory bowel disease	No	Yes	Yes	Yes	No	No	No	No	No
Metformin		Diabetes	Yes	Yes	Yes	Yes	Yes	Yes	Yes	Yes	Yes
Methazolamide		Antiglaucoma, diuretic	No	Yes	No	No	No	No	Yes	No	Yes
Methimazole	Thiamazole	Hyperthyroidism	No	Yes	No	No	Yes	No	Yes	No	Yes
Methylnaltrexone		Opioid-induced constipation	No	No	Yes	Yes	No	Yes	No	No	Yes
Metoclopramide		Anti-emesis	Yes	Yes	Yes	Yes	No	No	Yes	Yes	Yes
Midazolam		Sedation	Yes	Yes	Yes	Yes	No	No	No	No	No
Mifepristone		Abortifacient	Yes	Yes	Yes	Yes	Yes	No	Yes	Yes	Yes
Minocycline		Antibiotic	No	Yes	Yes	Yes	No	No	Yes	Yes	Yes
Mirtazapine		Depression	No	Yes	Yes	Yes	No	No	No	No	No
Mometasone	Mometasone furoate	Asthma prophylaxis	No	Yes	Yes	No	No	No	No	No	No
Montelukast		Allergies	No	Yes	Yes	Yes	No	No	No	No	No
Mycophenolate	Mycophenolic acid	Immunosuppressant	No	Yes	Yes	Yes	Yes	No	No	No	Yes
Nadroparin		Prophylaxis of venous thromboembolism	No	Yes	Yes	Yes	No	Yes	Yes	No	Yes
Naftopidil		Benign prostatic hyperplasia	No	Unclear	Yes	Yes	Yes	Yes	Yes	No	Yes
Naltrexone		Opioid receptor antagonist	No	Yes	Yes	Yes	Yes	No	Yes	No	Yes
Naproxen		Analgesia	No	Yes	Yes	Yes	Yes	No	Yes	Yes	Yes
Nelfinavir		Anti-retroviral	No	No	Yes	Yes	No	No	Yes	Yes	Yes
Niclosamide		Parasitic infection	Yes	Yes	Yes	Yes	No	No	Yes	No	Yes
Nicotinamide	Niacinamide	Niacin Deficiency, Skin cancer chemoprevention	Yes	Yes	Yes	Yes	No	No	Yes	Yes	Yes
Nifedipine		Hypertension	Yes	Yes	Yes	Yes	Yes	No	Yes	Yes	Yes
Nifurtimox		Chagas disease, African sleeping sickness	Yes	Unclear	Yes	Yes	Yes	No	Yes	Yes	Yes
Nimodipine		Hypertension	No	Yes	Yes	Yes	No	No	No	No	No
Nisoldipine		Hypertension	No	Yes	Yes	Yes	No	No	No	No	No
Nitazoxanide		Anti-protozoal	No	Yes	Yes	Yes	No	No	No	No	No
Nitisinone		Hereditary tyrosinemia type 1	No	No	No	No	Yes	No	No	No	Yes
Nitroglycerin	Glyceryl trinitrate	Nitro-vasodilator	Yes	Yes	Yes	Yes	Yes	No	Yes	Yes	Yes
Nitroxoline		Antibiotic	No	Yes	Yes	Yes	No	No	Yes	No	Yes
Norethandrolone		Aplastic anaemia	No	Yes	Yes	Yes	No	No	Yes	Yes	Yes
Noscapine		Anti-tussive	No	Yes	Yes	Yes	No	No	Yes	No	Yes
Olanzapine		Psychotic disorders	No	Yes	Yes	Yes	No	No	No	No	No
Olmesartan		Hypertension	No	Yes	Yes	Yes	No	No	No	No	No
Olsalazine		Rheumatoid arthritis; ulcerative colitis; active Crohn’s Disease.	No	Yes	Yes	Yes	No	No	No	No	No
Omega 3	Lovaza, Fish Oil, Omacor, eicosapentaenoic acid, docosahexaenoic acid	Hyperlipidaemia	No	Unclear	Yes	Yes	Yes	Yes	Yes	Yes	Yes
Omeprazole		Antacid	Yes	Yes	Yes	Yes	Yes	No	Yes	No	Yes
Orlistat		Obesity	No	Yes	Yes	Yes	No	No	No	No	No
Ormeloxifene		Contraceptive	No	Yes	Yes	Yes	No	No	No	No	No
Oseltamivir		Anti-viral	Yes	Yes	Yes	Yes	No	No	No	No	No
Ouabain		Cardiac arrhythmia	No	Yes	Yes	Yes	No	No	No	No	No
Oxcarbazepine		Epilepsy	No	Yes	Yes	No	No	Yes	No	No	Yes
Pamidronic acid	Pamidronate	Osteoporosis	No	Yes	Yes	Yes	Yes	No	Yes	Yes	Yes
Pantoprazole		Antacid	No	Yes	Yes	Yes	Yes	Yes	Yes	Yes	Yes
Paricalcitol	Vitamin D2	Hyperparathyroidism	No	Yes	Yes	Yes	Yes	No	Yes	Yes	Yes
Penfluridol		Psychotic disorders	No	Yes	Yes	Yes	No	No	No	No	No
Pentamidine		Parasitic infection	Yes	Yes	Yes	Yes	No	No	Yes	No	Yes
Pentoxifylline		Peripheral artery disease	No	Yes	Yes	Yes	No	No	Yes	Yes	Yes
Perphenazine		Psychotic disorders	No	Yes	Yes	Yes	No	No	No	No	No
Phenoxybenzamine		Pheochromocytoma	No	Yes	Yes	Yes	No	No	No	No	No
Phentolamine		Vasodilator	No	Yes	Yes	Yes	No	No	No	No	No
Phenylbutyrate	Glycerol Phenylbutyrate	Urea cycle disorders	No	Unclear	Yes	Yes	Yes	No	Yes	Yes	Yes
Phenytoin		Epilepsy	Yes	Yes	Yes	Yes	No	No	No	No	No
Pimozide		Psychotic disorders	No	Yes	Yes	Yes	Yes	No	No	No	Yes
Pioglitazone		Diabetes	No	Yes	Yes	Yes	Yes	No	Yes	Yes	Yes
Pirfenidone		Anti-fibrotic	No	No	Yes	Yes	No	No	Yes	No	Yes
Piroxicam		Analgesia	No	Yes	Yes	Yes	Yes	No	Yes	Yes	Yes
Plerixafor	AMD3100	Autologous HSCT	No	No	Yes	Yes	No	No	Yes	Yes	Yes
Pravastatin		Hyperlipidaemia	No	Yes	Yes	Yes	No	Yes	Yes	Yes	Yes
Prazosin		Hypertension	No	Yes	Yes	Yes	No	No	No	No	No
Prochlorperazine	Prochlorperazine dimaleate	Psychotic disorders	No	Yes	Yes	Yes	No	No	Yes	Yes	Yes
Promethazine		Psychotic disorders	No	Yes	Yes	Yes	No	No	No	No	No
Propafenone		Anti-arrhythmic	No	Yes	Yes	Yes	No	No	No	No	No
Propranolol		Hypertension	Yes	Yes	Yes	Yes	Yes	Yes	Yes	Yes	Yes
Pyridoxine	Vitamin B6	Vitamin B6 deficiency	Yes	Yes	Yes	Yes	No	No	Yes	Yes	Yes
Pyrimethamine		Parasitic infection	Yes	Yes	Yes	Yes	Yes	No	Yes	No	Yes
Pyrvinium Pamoate	Pyrvinium	Parasitic infection	No	Yes	Yes	Yes	No	No	No	No	No
Quetiapine		Psychotic disorders	No	Yes	Yes	No	No	No	No	No	No
Rabeprazole		Antacid	No	Yes	Yes	Yes	No	No	Yes	No	Yes
Ranitidine		Antacid	Yes	Yes	Yes	Yes	Yes	No	Yes	Yes	Yes
Ranolazine		Anti-angina	No	No	Yes	Yes	No	No	No	No	No
Repaglinide		Diabetes	No	No	Yes	Yes	No	No	No	No	No
Ribavirin		Anti-viral	Yes	Yes	Yes	Yes	Yes	No	Yes	Yes	Yes
Rifabutin		Antibiotic	Yes	Yes	Yes	No	No	No	No	No	No
Riluzole		ALS	No	Yes	Yes	Yes	No	No	Yes	Yes	Yes
Risperidone		Psychotic disorders	Yes	Yes	Yes	Yes	No	No	No	No	No
Ritonavir		Anti-retroviral	Yes	No	Yes	Yes	Yes	No	Yes	Yes	Yes
Roflumilast		COPD	No	No	Yes	Yes	No	No	Yes	No	Yes
Rosuvastatin		Hyperlipidaemia	No	Yes	Yes	Yes	No	No	Yes	Yes	Yes
Roxithromycin		Bacterial infections	No	Yes	Yes	Yes	Yes	No	No	No	Yes
Sertraline		Depression	No	Yes	Yes	Yes	No	No	Yes	Yes	Yes
Sildenafil		Erectile dysfunction	No	Yes	Yes	Yes	Yes	No	Yes	No	Yes
Simvastatin		Hyperlipidaemia	Yes	Yes	Yes	Yes	No	Yes	Yes	Yes	Yes
Sirolimus	Rapamycin	Inhibit organ transplant rejection	No	Yes	Yes	Yes	Yes	No	Yes	Yes	Yes
Sodium Aurothiomalate		Active progressive rheumatoid arthritis	No	Yes	Yes	Yes	No	No	Yes	Yes	Yes
Sodium Bicarbonate		Relief of wind and griping pains	No	Yes	Yes	Yes	Yes	No	Yes	Yes	Yes
Spironolactone		Congestive cardiac failure, Hepatic cirrhosis with ascites and oedema, Malignant ascites, Nephrotic syndrome, Diagnosis and treatment of primary aldosteronism.	Yes	Yes	Yes	Yes	No	No	Yes	Yes	Yes
Sulfasalazine		Rheumatoid arthritis; ulcerative colitis; active Crohn’s Disease.	Yes	Yes	Yes	Yes	Yes	No	Yes	Yes	Yes
Sulindac		Relief of signs and symptoms of osteoarthritis, rheumatoid arthritis, ankylosing spondylitis, acute painful shoulder (acute subacromial bursitis/supraspinatus tendinitis), and acute gouty arthritis.	No	Yes	Yes	Yes	No	Yes	Yes	Yes	Yes
Tadalafil		Erectile dysfunction	No	No	Yes	Yes	No	No	Yes	Yes	Yes
Telmisartan		Hypertension, cardiovascular prevention	No	Yes	Yes	Yes	No	Yes	No	No	Yes
Terbinafine		Treatment of tinea pedis (athlete’s foot), tinea cruris (dhobie (jock) itch) and tinea corporis (ringworm)	Yes	Yes	Yes	Yes	No	No	Yes	No	Yes
Thiabendazole		Parasitic infection	No	Unclear	Yes	Yes	No	No	No	No	No
Thioridazine		Psychotic disorders	No	Yes	Yes	Yes	Yes	No	Yes	No	Yes
Ticagrelor		Prevention of atherothrombotic events in combo with ASA	No	No	Yes	Yes	No	No	Yes	No	Yes
Ticlopidine		Prevent strokes, blood loss	No	Yes	Yes	Yes	Yes	No	Yes	No	Yes
Tigecycline		Infections	No	No	Yes	Yes	No	No	Yes	No	Yes
Timolol		Hypertension, ischaemic heart disease, migraine	Yes	Yes	No	No	Yes	Yes	Yes	Yes	Yes
Tinzaparin		Prophylaxis of venous thromboembolism	No	No	Yes	Yes	No	No	Yes	No	Yes
Tioconazole		Parasitic infection	No	Yes	Yes	Yes	No	No	No	No	No
Tocilizumab		Rheumatoid arthritis	No	No	Yes	Yes	No	No	Yes	Yes	Yes
Tofacitinib		Rheumatoid arthritis	No	No	Yes	Yes	Yes	No	No	No	Yes
Tolfenamic Acid	Tolfenamate	Migraine	No	Yes	Yes	Yes	No	No	No	No	No
Topiramate		Epilepsy (tonic clonic seizure)	No	Yes	Yes	Yes	No	No	No	No	No
Tranexamic Acid		Blood loss - fibrinolysis	Yes	Yes	Yes	Yes	Yes	No	Yes	Yes	Yes
Trazodone		Depression, anxiety	No	No	Yes	Yes	No	No	No	No	No
Triamterene		Diuretic, oedema in cardiac failure, cirrhosis of the liver or the nephrotic syndrome, and in that associated with corticosteroid treatment	No	Yes	Yes	Yes	No	No	No	No	No
Trifluoperazine		Psychotic disorders	No	No	Yes	Yes	No	No	Yes	No	Yes
Trimetazidine		Angina pectoris	No	Yes	Yes	Yes	No	No	Yes	No	Yes
Ulinastatin		Severe sepsis & pancreatitis	No	Unclear	Yes	Yes	No	No	Yes	Yes	Yes
Valproic Acid	Valproate	Epilepsy	Yes	Yes	Yes	Yes	Yes	Yes	Yes	Yes	Yes
Valsartan		Hypertension, myocardial infarction, heart failure	No	Yes	Yes	Yes	No	Yes	No	No	Yes
Vardenafil		Erectile dysfunction	No	No	Yes	Yes	No	No	Yes	No	Yes
Verapamil		Hypertension, angina pectoris	Yes	Yes	Yes	Yes	Yes	No	Yes	Yes	Yes
Verteporfin		Exudative age-related macular degeneration	No	No	Yes	Yes	Yes	No	Yes	Yes	Yes
Warfarin		Prophylaxis of systemic embolism, of venous thrombosis and pulmonary embolism.	Yes	Yes	Yes	Yes	No	Yes	Yes	Yes	Yes
Zidovudine	Azidothymidine	Anti-retroviral	Yes	Yes	Yes	Yes	Yes	No	Yes	Yes	Yes
Zoledronic Acid	Zoledronate	Osteoporosis, prophylaxis of skeletal fractures and treat hypercalcaemia of malignancy, treat pain from bone metastases	Yes	Yes	Yes	Yes	Yes	Yes	Yes	Yes	Yes
